# Decrease in CD14++CD16+ Monocytes in Low-Immunological-Risk Kidney Transplant Patients with Subclinical Borderline Inflammation

**DOI:** 10.3390/jcm10215051

**Published:** 2021-10-28

**Authors:** Abelardo Caballero, Teresa Vazquez-Sanchez, Pedro Ruiz-Esteban, Myriam Leon, Juana Alonso-Titos, Veronica Lopez, Eugenia Sola, Elena Gutierrez, Mercedes Cabello, Cristina Casas-Gonzalez, Rafael Pozo-Alvarez, Juan Delgado-Burgos, Domingo Hernandez

**Affiliations:** 1 Immunology Department, Instituto de Investigación Biomédica de Málaga (IBIMA), Hospital Universitario Regional de Málaga, Universidad de Málaga, 29010 Malaga, Spain; abelardo.caballero.sspa@juntadeandalucia.es; 2 Nephrology Department, Instituto de Investigación Biomédica de Málaga (IBIMA), Hospital Universitario Regional de Málaga, Universidad de Málaga, 29010 Malaga, Spain; teresavs89@hotmail.com (T.V.-S.); pedro_ruiz_esteban@hotmail.com (P.R.-E.); juana12041988@hotmail.com (J.A.-T.); verolopezjim@yahoo.es (V.L.); sola.moyano.eugenia@gmail.com (E.S.); elenaguvi@hotmail.com (E.G.); mcabello82@hotmail.com (M.C.); cristinacasasgonzalez@gmail.com (C.C.-G.); rafa94pa@gmail.com (R.P.-A.); juandelgadob@gmail.com (J.D.-B.); 3 Pathology Department, Instituto de Investigación Biomédica de Málaga (IBIMA), Hospital Universitario Regional de Málaga, Universidad de Málaga, 29010 Malaga, Spain; mlfradejas@gmail.com

**Keywords:** kidney transplant, monocytes, CD14++CD16+, kidney biopsy, borderline lesions

## Abstract

We determined the association between CD14++CD16+ monocytes and subclinical infiltrates that do not reach the histological threshold for rejection (≥Banff IA). We studied low-immunological-risk kidney-transplant recipients in a clinical trial (NCT02284464; EudraCT 2012-003298-24) whose protocol biopsy in the third month showed no significant changes or borderline lesions (BL). Flow cytometry was used to analyze the percentage of CD14++CD16+ monocytes in peripheral blood (PB) and blood from a fine-needle-aspiration biopsy (FNAB). A protocol biopsy was performed in 81 low-immunological-risk patients, of whom 15 were excluded (BK polyomavirus and rejection). The 28 (42.4%) with borderline lesions had significantly low levels of CD14++CD16+ in PB compared to patients with normal biopsies (7.9 ± 5.4 vs. 13.0 ± 12.8; *p* = 0.047). Patients without significant changes had similar percentages of CD14++CD16+ monocytes in the graft blood (GB) and FNAB blood. The percentage of these monocytes in the patients with an interstitial infiltrate, however, increased significantly in the FNAB blood compared to the GB: 16.9 ± 16.6 vs. 7.9 ± 5.4; *p* = 0.006. A difference of 50% in CD14++CD16+ in the GB versus the PB was a significant risk factor (*p* = 0.002) for BL, increasing the risk seven times. A decrease in CD14++CD16+ in the PB could be associated with the recruitment of these cells to the graft tissue in cases of subclinical BL inflammatory infiltrates below the threshold for rejection.

## 1. Introduction

Although monocytes/macrophages, together with T lymphocytes, have been recognized since 1958 as the two main cell types in the infiltration of grafts with acute rejection [1], research on the role of the myeloid lineage has not kept pace. Nonetheless, as evidence suggests a crucial role for monocytes/macrophages in the pathogenesis of rejection, associated with a worse result in terms of survival [2], interest has now increased in this type of cell lineage. 

Monocytes can be subdivided into three subpopulations that are phenotypically and functionally different depending on the expression of the lipopolysaccharide receptor, CD14, and the Fcγ III receptor, CD16. In healthy people, about 80–90% of monocytes are highly positive for CD14 and negative for CD16 (CD14++CD16−) classical monocytes. The remaining 10–20% of monocytes are CD16 positive, and can be subdivided into CD14++CD16+ and CD14+CD16++ cells, intermediate and non-classical monocytes, respectively [3]. The CD16+ monocytes are considered proinflammatory due to the production of proinflammatory cytokines, like TNF-α and IL-1β, in comparison with classical monocytes [4]. 

By definition, subclinical inflammatory graft histological lesions, especially borderline lesions (BL), refer to the presence of minor degrees of inflammation in the kidney biopsy in the absence of impaired allograft function. Some transplant centers use protocol biopsies at predetermined times to detect early subclinical rejection before clinical dysfunction [5,6,7]. Protocol biopsies can detect multiple phenotypes of subclinical inflammation (SCI), including subclinical T cell-mediated borderline rejection (SC-B-TCMR), subclinical T cell-mediated acute rejection (SC-TCMR) and subclinical antibody-mediated rejection (SC-AMR). Recent studies suggest that early detection of subclinical rejection can identify patients at greater risk of graft failure and who might thus benefit from greater exposure to immunosuppression [8,9]. However, subclinical infiltrates that do not reach the histological threshold for rejection have received less attention.

In the present study we quantified using flow cytometry the percentage of CD14++CD16+ monocytes in both a sample of venous blood and in a blood sample obtained by a fine-needle-aspiration biopsy (FNAB). This technique is rapid and less invasive than a kidney biopsy and provides useful information about the immunological status of the graft [10]. We have determined whether there is an association between the intermediate monocytes and borderline subclinical lesions that fail to reach the threshold for rejection in low-immunological-risk patients.

## 2. Materials and Methods

### 2.1. Study Design

The study included those patients in the independent multicenter clinical trial “Steroid Withdrawal and Novo Donor-specific Anti-HLA Antibodies in Renal Transplant Patients: a Prospective, Randomized and Controlled Study in Parallel Groups” (NCT02284464; EudraCT 2012-003298-24) who received a KT at the Regional University Hospital of Malaga (Spain) between March 2015 and December 2016 and who had a follow-up at the protocol biopsy in the third month post-transplant. The inclusion and exclusion criteria were those used for the mentioned clinical trial [11,12]. 

Written informed consent was obtained from all the participants in the clinical trial. The review of their medical records was undertaken in accordance with Spanish law and the clinical trial was approved by the Malaga Ethics Committee and the Spanish Agency of Medicines and Medical Devices. Likewise, the clinical trial was registered in Clinical Trials with the number NCT02284464 (https://clinicaltrials.gov, accessed on 28 October 2021), in the Spanish Registry of Clinical Trials (REEC) (https://reec.aemps.es/reec/public/web.html, accessed on 28 October 2021) and the European Union Drug Regulating Authorities Clinical Trials Database (EudraCT) with the number 2012-003298-24 (https://eudract.ema.europa.eu, accessed on 28 October 2021). At all times the standards of Good Clinical Practice and the Helsinki and Istanbul declarations were followed.

### 2.2. Data Collection

The following baseline data were collected: age, sex, weight, donor type, renal replacement therapy, cause of end stage kidney disease, induction therapy, cold ischemia time, pretransplant transfusions, pretransplant PRA, number of incompatibilities, and delayed graft function.

Other data recorded at the time of the protocol biopsy included creatinine, proteinuria, blood pressure, and levels and dose of tacrolimus.

The histological scores of the protocol biopsies were recorded according to the Banff 2017 classification: glomerulitis (g); peritubular capillaritis (ptc); tubulitis (t); interstitial inflammation (i); arteritis (v); interstitial fibrosis (ci); tubular atrophy (ct); transplant glomerulopathy (cg); chronic vascular lesions (cv); and arteriolar hyalinosis (ah).

### 2.3. Fine-Needle-Aspiration Biopsy

**Figure 1:**
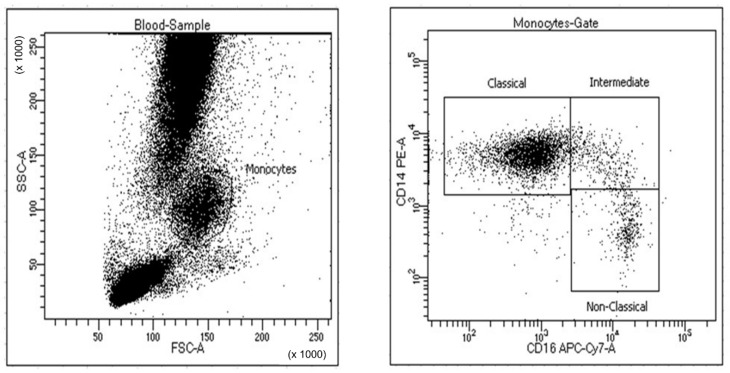
Flow cytometry gating for monocyte CD14++CD16+ cells from a sample obtained by FNAB.

All the patients provided informed consent for an FNAB that was done just before taking the biopsy from conventional tissue. To evaluate the aspirates from the FNAB, 66 patients were classified as apt, that is, they had sufficient blood content so that all the FNAB samples were considered adequate for flow cytometry analysis. On the graph of complexity versus size the FNAB samples presented a similar leukocyte distribution to that seen in the peripheral blood (Figure 1), with analysis of a mean of 8200 cells on the monocyte gate. All the samples were collected without any patient complications.

The FNAB was performed by ultrasound-guided puncture (SonoSite Fujifilm, Sitelink Image Manager 2.2, Amsterdam, The Netherlands), without anesthesia, at the external cortical pole of the graft (upper or lower), thus avoiding the large vessels, using a 0.8 mm/25 G needle mounted in a Cameco gun. Two FNABs were performed per patient and the aspirates were grouped before further analysis. If the gross evaluation showed that an aspirate had predominantly no blood another sample was taken immediately. Only samples with a predominance of blood were processed for flow cytometry analysis. This material was placed in 10 mL of HEPES culture medium buffered with heparin and human serum albumin. The sample was centrifuged at 1600 rpm and the pellet, resuspended in 50 µL, was added to the tube for analysis.

### 2.4. Flow Cytometry Analysis of the CD14++CD16+ Monocyte Subpopulation

Venous blood was collected for fluorescence-activated cell sorter (FACS) analysis at three months post-transplantation at the same time as the biopsy. The FACS analysis was performed using a FACSCanto flow cytometer (Becton Dickinson, Iberia) that was calibrated with beads labeled with the relevant two fluorochromes. We used mouse monoclonal antibodies directly conjugated to phycoerythrin (PE) and allophycocyanin–cyanine 7 (APC-Cy7-A): CD14-PE (mouse anti-human CD14 BD IS) and CD16-APC-Cy7-A (mouse anti-human CD16 BD IS). For assays we used one tube (BD Falcon 5 mL Polystyrene Round-Bottom Tube 12 75 mm style) and we added 50 µL of the anticoagulated blood sample and 10 µL of each monoclonal antibody: CD14 and CD16. The labeled isotype control antibodies were used at equivalent concentrations. After incubation for 20 min the sample was lysed with BD FACS Lysing Solution for 15 min. It was later centrifuged at 1600 rpm and the pellet was resuspended in 200 µL of PBS for analysis. 

The monocyte populations were gated in the SSC/FSC (Figure 1). The intermediate polygon gate was selected to calculate the percentage of CD14++CD16+ monocytes (Figure 1). The gating and analyses were performed using BD FACSDiva software v 6.x, available atBD FACSDiva™ Software|BD Biosciences (28 October 2021).

The FACS sample obtained was processed identically to the venous blood sample.

### 2.5. Outcome

The outcome variable was the percentage change in the monocyte subpopulation CD14++CD16+ in the graft blood (GB) extracted by an FNAB as compared to the peripheral venous blood (PB) ((GB value − PB value) × 100/PB value). As no previous studies exist, we determined differences in the CD14++CD16+ monocytes in the GB versus the PB of 10%, 20%, 30%, 40%, 50%, and 75% in order to define the best cut point to discriminate between biopsies with no significant changes and biopsies with borderline lesions. 

### 2.6. Statistical Analysis

A descriptive analysis of the results was undertaken, expressing the quantitative variables as the mean ± standard deviation and the qualitative variables as relative percentages. Normality was tested (using the Kolmogorov–Smirnov and Shapiro–Wilk tests) to determine the distribution of the data. The hypothesis contrasts were tested using parametric or nonparametric tests (Student’s *t* or Mann–Whitney U tests).

A parsimonious binary logistic regression was done to determine the risk factors associated with the presence or absence of borderline lesions in the protocol biopsy.

We analyzed the sensitivity (the likelihood that a patient with borderline lesions in the protocol biopsy presents a certain difference in CD14++CD16+ monocytes in the GB versus the PB), the specificity (the likelihood that a patient without significant changes in the protocol biopsy does not present a certain difference in CD14++CD16+ monocytes in the GB versus the PB), and the positive and negative predictive values (the likelihood of having (positive predictive value) or not having (negative predictive value) borderline lesions once it is known whether that patient has or does not have a certain difference in the CD14++CD16+ monocytes) for the various differences in the monocyte subpopulation CD14++CD16+ in the GB versus the PB.

The equations are shown below:

Sensitivity = [(true positive/true positive + false negatives) × 100]

Specificity = [(true negative/true negative + false positives) × 100]

Positive predictive value = [(true positive/true positive + false positives) × 100]

Negative predictive value = [(true negative/true negative + false negatives) × 100]

The statistical analyses were performed with IBM SPSS Statistics V22.0 for Windows (IBM Corp., Armonk, NY, USA) and the significance was set at *p* < 0.05.

## 3. Results

### 3.1. Donor and Recipient Clinical and Demographic Characteristics

A protocol biopsy was performed on 81 low-immunological-risk patients in the third month post-transplant. For the analysis of CD14++CD16+ monocytes 15 of these patients were excluded for the following histological causes: two due to BK polyomavirus, three due to acute rejection IIA, four due to acute rejection IB, and six due to acute rejection IA. Thus, of the 66 patients finally analyzed, 38 (57.6%) had no significant alterations on the biopsy and 28 (42.4%) had a biopsy with BL according to the Banff 2017 classification [13]. 

Table 1 shows the baseline characteristics. All patients were treated with conventional triple therapy (tacrolimus + MMF/MFA + prednisone). The mean age was 53.9 ± 11.9 years and most were male (68.2%). The type of donor was deceased in 81.8%, and 58.5% of the recipients had hemodialysis as the replacement therapy. Induction treatment was given to 87.9% at the time of transplantation and the cold ischemia time was 11.4 ± 8.0 hours. Only 9.7% of the patients received transfusions before the transplant and 22.7% of the patients experienced delayed graft function.

Comparison between the patients with a protocol biopsy showing no significant changes and the patients with BL showed no significant differences for any of the variables studied, except for recipient age (*p* = 0.045), where the mean age of the patients with no significant changes was 57.8 ± 9.5 years versus 51.1 ± 12.8 years in the patients with BL. 

### 3.2. Histological Score of the Protocol Biopsy

As expected, the protocol biopsy score based on the Banff 2017 classification (score 0–3) [13] (Table 2) showed that tubulitis (1.0 ± 0.0 vs. 0.05 ± 0.23; *p* < 0.0001) and interstitial inflammation (1.0 ± 0.0 vs. 0.45 ± 0.50; *p* < 0.0001) were significantly greater in patients with BL compared to those showing no significant biopsy changes. The other parameters (glomerulitis, peritubular capillaritis, interstitial fibrosis, tubular atrophy, chronic vascular lesions, and arteriolar hyalinosis) were not significantly different, even though the score was higher in the group with borderline lesions. 

### 3.3. CD14++CD16+ Monocytes

Flow cytometry analysis of the CD14++CD16+ monocytes showed a significant difference in the PB between the group of patients with an interstitial infiltrate and those without (borderline 7.9 ± 5.4 vs. normal 13.0 ± 12.8; *p* = 0.047). To determine whether this reduction could be due to the recruitment of these monocytes to the graft interstitium they were also measured in the GB. The percentage of the monocytes was significantly greater than that in the PB (GB: 16.9 ± 16.6 vs. PB: 7.9 ± 5.4; *p* = 0.006). This difference, though, was not seen in the patients with no histological lesions (GB: 16.3 ± 14.3 vs. PB: 13.2 ± 12.9; *p* = 0.070).

### 3.4. Percentage Change of the CD14++CD16+ Monocytes

**Figure 2:**
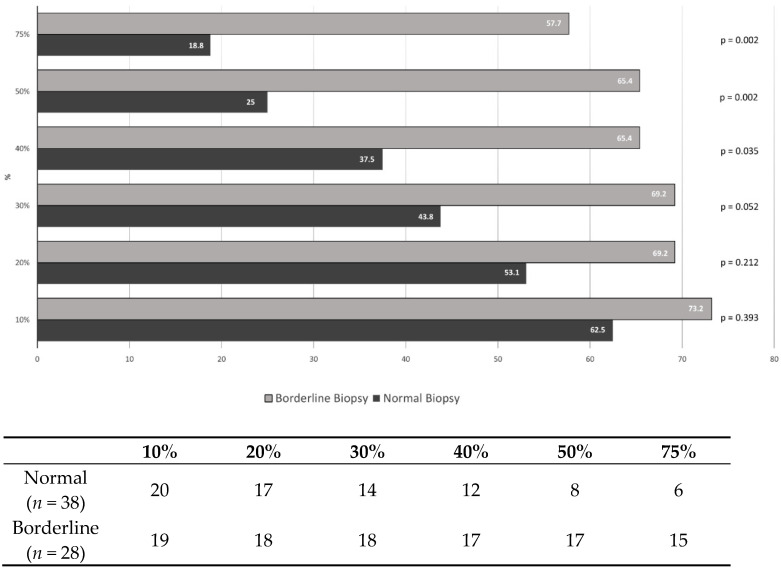
Difference in the CD14++CD16+ monocytes (Yes/No) in graft blood versus peripheral blood.

An analysis of the percentage change of the CD14++CD16+ monocytes in the GB versus the PB in the patients with and without BL is shown in Figure 2. A percentage difference of 40% in the GB versus the PB was associated with significant differences between the patients with and without BL (*p* = 0.035). These differences became more marked above 50% (*p* = 0.002); 65.4% of the patients with BL had a difference of 50% or more in the CD14++CD16+ monocyte population in the GB versus the PB. However, only 25% of the patients with no significant biopsy alterations showed this percentage change. 

For a percentage change of 50%, the sensitivity was 65.4% and specificity 75%. The positive and negative predictive values were 68% and 73%, respectively. For a percentage change of 75%, the sensitivity was 57.7%, specificity 81.3%, positive predictive value 70%, and negative predictive value 70%.

### 3.5. Risk Factors for Borderline Lesions

Table 3 shows the univariate and multivariate analysis of the binary logistic regression. The univariate analysis showed risk factors for borderline lesions to be a difference of 40% (OR 3.1 (1.1–9.3); *p* = 0.037), 50% (OR 5.7 (1.8–17.7); *p* = 0.003) and 75% (OR 5.9 (1.8–19.2); *p* = 0.003) and recipient age (OR 1.1 (1.0–1.1); *p* = 0.028).

The parsimonious multivariate analysis showed a risk factor for borderline lesions to be a difference in CD14++CD16+ in the GB versus the PB of 50% and 75%, as well as recipient age, increasing the risk seven times in the case of a rise in the monocytes and 7% per year for the recipient.

## 4. Discussion

The main finding of this study was that in recipients of a KT with a low-immunological risk and BL the level of CD16+ monocytes in the PB was significantly lower than in those patients with no histological alterations. Nevertheless, the monocyte count in the blood from the FNAB in patients with histological lesions was significantly increased compared to their level in the PB, a difference that was not seen in the patients with no histological lesions.

This finding could be related to an important presence of intermediate monocytes in the subclinical inflammatory infiltrate. This is the first study to use flow cytometry to examine the CD16+ monocyte population in association with BL in a group of patients with a normal biopsy and another group with subclinical inflammatory lesions.

An increase in CD16+ monocytes has been reported to be significantly associated with a high risk of acute rejection after a KT. Strangely, at the time of rejection a measurable reduction in the subset of circulating CD16+ monocytes paralleled the increase in CD16+ monocytes infiltrating the tissue, thus suggesting a migration towards the inflamed graft [14]. In our case, although the CD16+ monocytes were not studied in the biopsy specimen, we consider that a possible migration can be deduced if we consider the increase in these monocytes in the blood extracted by an FNAB, since an increase in leukocytes in capillary blood as compared to venous blood is associated with tissue inflammatory processes [15]. 

Samples contaminated with FNAB blood are normally discarded, mainly, when isolated interstitial inflammatory infiltration is assessed. However, we consider that, given that it comes from capillary blood and that the leukocyte count is similar to that in venous blood when no tissue inflammation exists [16], a significant increase in any subtype of leukocytes could be associated with their migration towards the inflamed tissue. Our results show that an increase of 50% or more in intermediate monocytes in graft blood compared to PB is indicative of the recruitment of these cells to the inflamed tissue. Although we have previously used this technique to study lymphocyte subpopulations [10], its use to aid a diagnosis requires confirmation with other trials.

Some borderline biopsies probably reflect early T cell-mediated rejection and progress to more severe lesions [17], while others simply reflect a mononuclear infiltrate with no definite diagnosis. As molecular studies have shown that most BL are not T cell-mediated, this type of histological lesion should not, therefore, be considered T cell-mediated rejection [18]. However, as we did not study the T cells, we do not know how many of our biopsies contained this type of cell. What we can say from our results, though, is that the CD14++CD16+ monocytes can be detected in most biopsies, such that this subtype of monocyte could be the predominant cell in the mononuclear infiltration of this lesion. However, it cannot be discarded that one or more of the other monocyte subtypes may play a relevant role. 

Studies of PB have documented a change towards the proinflammatory monocytes CD14++CD16+ in stable KT recipients and that this remains during the first six months post-transplant despite immunosuppressive therapy and a significant improvement in kidney function. These monocytes are able to produce IFNγ, which is a crucial step in the rejection process [5,19]. On the other hand, it has also been shown that the recognition of non-self by monocytes persists long after the acute surgical inflammation has diminished, indicating the important role of monocytes in the onset of long-term graft failure [20].

The monocyte and macrophage functions differ in the induction or maintenance of inflammation or fibrosis. Heart transplant rejection tissue studies have found an increase in the CD16+ monocytes and M2 macrophages, together with a reduction of these monocytes in PB [21]. Extrapolating these findings to our situation, we could speculate that the M2 macrophages, producers of IL10, would be continually increasing in the tissue during subclinical rejection and, together with the presence of the monocytes CD16+, with their presumably proinflammatory nature, would form part of the micro-environmental equilibrium of BL that could be followed by tissue fibrosis.

## 5. Conclusions

In summary, we consider that the subpopulation of intermediate monocytes could be relevant cells in subclinical borderline infiltrates, as they are significantly reduced in PB in transplant recipients with this type of histological lesion as compared to recipients who do not have these lesions. We think that the leukocyte count in blood from an FNAB could help explain this reduction and aid in the diagnosis. Additional prospective clinical trials are needed to confirm these findings. 

## Figures and Tables

**Table 1:** Clinical and demographic characteristics of the study patients.

	Total *n* = 66	Borderline (*n* = 28)	Normal (*n* = 38)	*p* Value
Donor age (years)	53.7 ± 11.3	55.3 ± 11.0	52.6 ± 11.6	0.357
Recipient age (years)	53.9 ± 11.9	57.8 ± 9.5	51.1 ± 12.8	0.045
Recipient weight (Kg)	76.9 ± 14.6	76.9 ± 14.6	76.9 ± 14.7	0.989
Donor sex (M) %	65.2	75.0	57.9	0.149
Recipient sex (M) %	68.2	67.9	68.4	0.961
Donor type (Deceased) %	81.8	89.3	76.3	0.177
Replacement therapy (HD) %	58.5	64.3	54.1	0.702
Cause of kidney disease (%)				
Glomerular	22.7	21.4	23.7	0.676
Diabetes	12.1	14.3	10.5	
Polycystosis	25.8	35.7	18.4	
Interstitial nephropathy	7.6	7.1	7.9	
Autoimmune	1.5	0.0	2.6	
Nephroangiosclerosis	7.6	3.6	10.5	
No determined	10.3	10.7	10.5	
Other	12.1	7.1	15.8	
Induction (%)	87.9	84.6	90.6	0.485
Cold ischemia time (hours)	10.7 ± 5.0	10.9 ± 3.7	10.6 ± 5.8	0.787
Transfusions (Yes) %	9.7	7.7	11.1	0.653
PRA pre-transplant (%)	0.2 ± 1.7	0.5 ± 2.6	0.0	0.326
Total HLA mismatches (*n*)	6.3 ± 2.5	6.6 ± 2.2	6.1 ± 2.7	0.425
HLA-DR mismatches (*n*)	1.2 ± 0.7	1.2 ± 0.6	1.2 ± 0.7	0.982
Delayed graft function (%)	22.7	25.0	21.1	0.705
Creatinine (mg/d)	1.6 ± 0.5	1.7 ± 0.5	1.6 ± 0.6	0.536
Proteinuria (mg/24 h)	268.7 ± 214.7	269.3 ± 239.8	268.2 ± 197.9	0.986
Diastolic blood pressure (mmHg)	71.6 ± 6.9	73.1 ± 6.0	70.8 ± 7.3	0.280
Systolic blood pressure (mmHg)	125.9 ± 11.5	124.8 ± 9.3	126.4 ± 12.6	0.639
Tacrolimus levels (ng/mL)	9.7 ± 2.4	9.3 ± 2.6	9.9 ± 2.2	0.290

**Table 2:** Histological biopsy score in the third month.

	Total (*n* = 66)	Borderline (*n* = 28)	Normal (*n* = 38)	*p* Value
g (0–3)	0.11 ± 0.31	0.14 ± 0.36	0.08 ± 0.27	0.412
ptc (0–3)	1.25 ± 0.49	0.27 ± 0.72	0.03 ± 0.16	0.104
t (0–3)	0.46 ± 0.50	1.0 ± 0.0	0.05 ± 0.23	0.000
i (0–3)	0.68 ± 0.47	1.0 ± 0.0	0.45 ± 0.50	0.000
v (0–3)	0.0	0.0	0.0	
ci (0–3)	0.49 ± 0.50	0.57 ± 0.50	0.42 ± 0.50	0.233
ct (0–3)	0.39 ± 0.49	0.50 ± 0.51	0.31 ± 0.47	0.139
cg (0–3)	0.0	0.0	0.0	
cv (0–3)	0.47 ± 0.61	0.54 ± 0.69	0.42 ± 0.55	0.457
ah (0–3)	0.36 ± 0.62	0.50 ± 0.75	0.26 ± 0.50	0.153
ct + ci	0.88 ± 0.95	1.07 ± 0.98	0.74 ± 0.92	0.160
ct + ci + cg + cv	1.35 ± 1.23	1.61 ± 1.40	1.16 ± 1.08	0.162

Abbreviations. ah: arteriolar hyalinosis; ci: interstitial fibrosis; cg: graft glomerulopathy; ct: tubular atrophy; cv: chronic vascular lesions; g: glomerulitis; i: interstitial inflammation; ptc: peritubular capillaritis; t: tubulitis; v: arteritis.

**Table 3:** Logistic regression to determine risk factors associated with the appearance of borderline lesions in the protocol biopsy.

Univariate Analysis			Multivariate Analysis			
	OR (95% CI)	*p*	OR (95% CI)	*p*	OR (95% CI)	*p*
Difference of 10%	1.629 (0.529–5.011)	0.395				
Difference of 20%	1.985 (0.671–5.871)	0.215				
Difference of 30%	2.893 (0.976–8.578)	0.055				
Difference of 40%	3.148 (1.070–9.264)	0.037				
Difference of 50%	5.667 (1.818–17.667)	0.003	7.325 (2.079–25.807)	0.002		
Difference of 75%	5.909 (1.815–19.238)	0.003			7.708 (2.096–28.351)	0.002
Sex (M)	0.974 (0.342–2.777)	0.961				
Recipient age (years)	1.054 (1.006–1.105)	0.028	1.071 (1.010–1.135)	0.022	1.069 (1.011–1.131)	0.020
Delayed graft function (Yes/No)	1.250 (0.393–3.978)	0.706				
Cold ischemia time (hours)	1.014 (0.913–1.125)	0.796				
Transfusions (Yes/No)	0.667 (0.113–3.945)	0.655				
Donor age (years)	1.021 (0.977–1.068)	0.352				
Induction therapy (Yes/No)	0.569 (0.115–2.807)	0.489				
Total HLA mismatches (*n*)	1.088 (0.886–1.337)	0.420				
HLA–DR mismatches (*n*)	1.009 (0.483–2.106)	0.982				
Creatinine	1.352 (0.528–3.464)	0.530				
Proteinuria	1.000 (0.997–1.003)	0.986				
Tacrolimus levels	0.887 (0.712–1.106)	0.287				

## Data Availability

Data available upon request due to restrictions of privacy. The data presented in this study are available upon request from the corresponding author. The data are not publicly available due to compliance with Organic Law 15/1999.
